# Minimizing invasiveness and simplifying the surgical procedure for upper and middle early gastric cancer with near-infrared light and organ traction

**DOI:** 10.1186/s12957-023-02960-8

**Published:** 2023-03-07

**Authors:** Shinnosuke Nagano, Yuki Ushimaru, Ryohei Kawabata, Akihiro Kitagawa, Nobuyoshi Ohara, Yuichiro Miyake, Hideo Tomihara, Sakae Maeda, Shingo Noura, Atsushi Miyamoto, Kazuhiro Nishikawa

**Affiliations:** Department of Gastroenterological Surgery, Sakai City Medical Center, 1-1-1 Ebaraji-Cho, Nishi-Ku, Sakai City, Osaka, 593-8304 Japan

**Keywords:** Gastric cancer, Upper and middle body of the stomach, Laparoscopic distal gastrectomy, Billroth I reconstruction

## Abstract

**Background:**

Surgeons are often faced with optimal resection extent and reconstructive method problems in laparoscopic gastrectomy for gastric cancer in the upper and middle body of the stomach. Indocyanine green (ICG) marking and Billroth I (B-I) reconstruction were used to solve these problems with the organ retraction technique.

**Case presentation:**

A 51-year-old man with upper gastrointestinal endoscopy revealed a 0-IIc lesion in the posterior wall of the upper and middle gastric body 4 cm from the esophagogastric junction. Clinical T1bN0M0 (clinical stage IA) was the preoperative diagnosis. Laparoscopic distal gastrectomy (LDG) and D1 + lymphadenectomy was decided to be performed considering postoperative gastric function preservation. The ICG fluorescence method was used to determine the accurate tumor location since the determination was expected to be difficult to the extent of optimal resection with intraoperative findings. By mobilizing and rotating the stomach, the tumor in the posterior wall was fixed in the lesser curvature, and as large a residual stomach as possible was secured in gastrectomy. Finally, delta anastomosis was performed after increasing gastric and duodenal mobility sufficiently. Operation time was 234 min and intraoperative blood loss was 5 ml. The patient was allowed to be discharged on postoperative day 6 without complications.

**Conclusion:**

The indication for LDG and B-I reconstruction can be expanded to cases where laparoscopic total gastrectomy or LDG and Roux-en-Y reconstruction has been selected for early-stage gastric cancer in the upper gastric body by combining preoperative ICG markings and gastric rotation method dissection.

**Supplementary Information:**

The online version contains supplementary material available at 10.1186/s12957-023-02960-8.

## Background

Gastric cancer surgical treatment in the upper and middle third of the stomach near the esophagogastric junction (EGJ), and it is difficult to determine the extent of the resection margin and reconstruction method. Our hospital is actively selecting laparoscopic distal gastrectomy (LDG) for upper and middle body gastric cancer considering postoperative gastric function. Billroth I (B-I) reconstruction with the delta-shaped anastomosis is the first choice for reconstruction methods from the viewpoint of procedure simplicity, shortened operation time, and reducing postoperative complications. The surgical and reconstructive procedures depend significantly on the size of the remaining stomach in the case of upper and middle gastric cancer, so the determination of the tumor dissection line and the stapling method was critical. We used Indocyanine green (ICG) marking and a method of rotating the stomach for stapling for early upper and middle posterior wall gastric cancer. A successful LDG and B-I reconstruction was performed.

## Case presentation

**Fig. 1:**
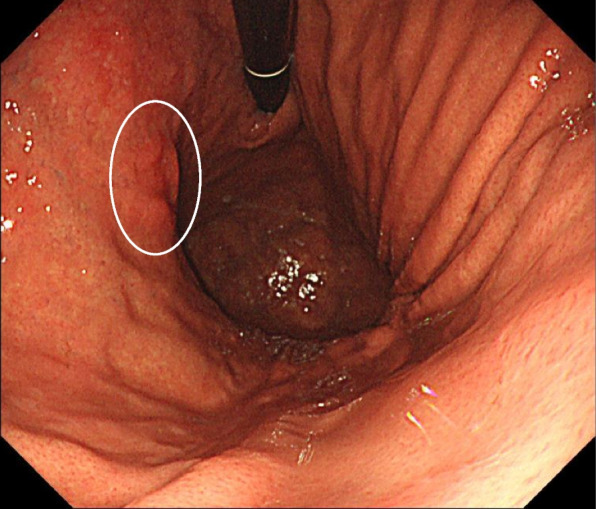
Upper gastrointestinal endoscopy. Upper gastrointestinal endoscopy examination revealed a 0-IIc lesion in the posterior wall of the upper and middle gastric body 4 cm from the EGJ

A 51-year-old man with an upper gastrointestinal endoscopy examination revealed a 0-IIc lesion in the posterior wall of the upper and middle gastric body 4 cm from the EGJ (Fig. 1). The patient was diagnosed with gastric cancer clinical T1bN0M0 (clinical stage IA) and decided on surgical treatment.

The patient had early-stage gastric cancer, and LDG and D1 + lymphadenectomy performance was planned to preserve the remaining stomach. It would be difficult to determine the intraoperative dissection margin by laparoscopic operation. ICG fluorescence marking was performed before surgery because of this. A 100-fold diluted, 0.5 ml ICG was injected at the just oral side of the tumor 1 day preoperatively. B-I reconstruction with the delta-shaped anastomosis was planned first as the reconstruction method.

### Surgical procedure

An additional movie file shows this surgical procedure in more detail (see Additional file 1).

#### Lymphadenectomy and mobilization

**Fig. 2:**
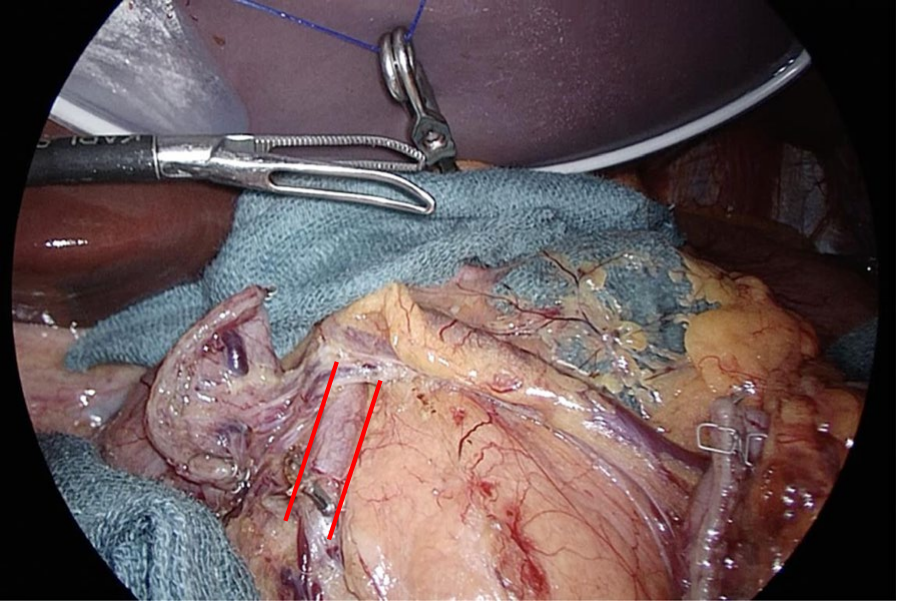
Surgical procedure: lymphadenectomy and mobilization. The posterior duodenal wall was fully mobilized while exposing and confirming the gastroduodenal artery (red line)

Lymph node dissection was performed similarly to that of conventional LDG and D1 + lymphadenectomy. The posterior duodenal wall was fully mobilized while exposing and confirming the gastroduodenal artery when performing lymph node No. 6 dissection (Fig. 2). The posterior wall of the fundus was also mobilized since it was a case with a high possibility of becoming a tiny remnant stomach.

#### Stapling the oral side of the tumor

**Fig. 3:**
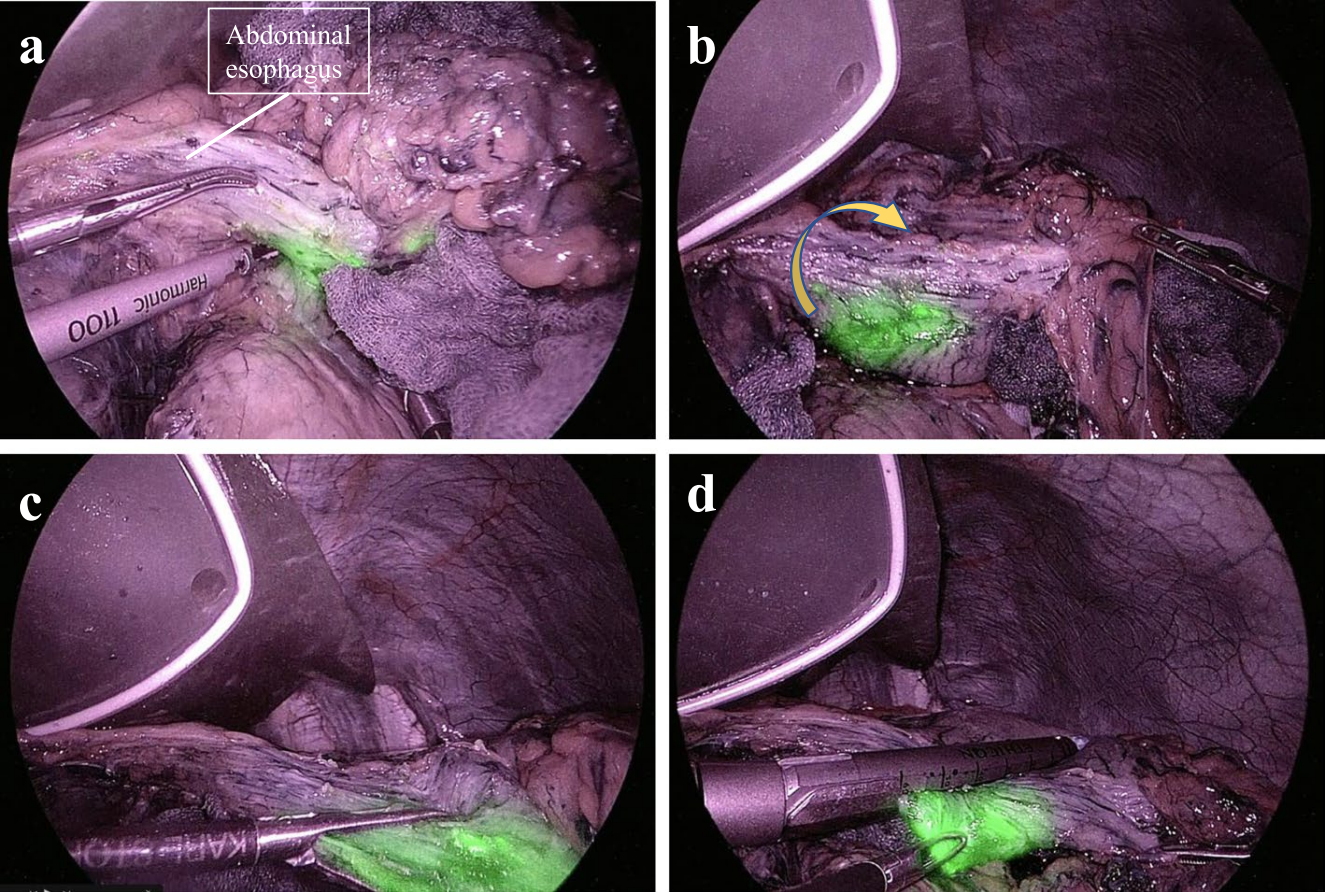
Surgical procedure: stapling the oral side of the tumor. **a** The tumor was located on the posterior wall of the upper body of the stomach by using an IR laparoscope camera. **b**, **c** The tumor in the posterior wall was fixed in the lesser curvature by rotating the stomach clockwise (yellow arrow). **d** First stapling. A 60-mm stapler was used from the lesser curvature side to ensure a 2-cm margin

**Fig. 4:**
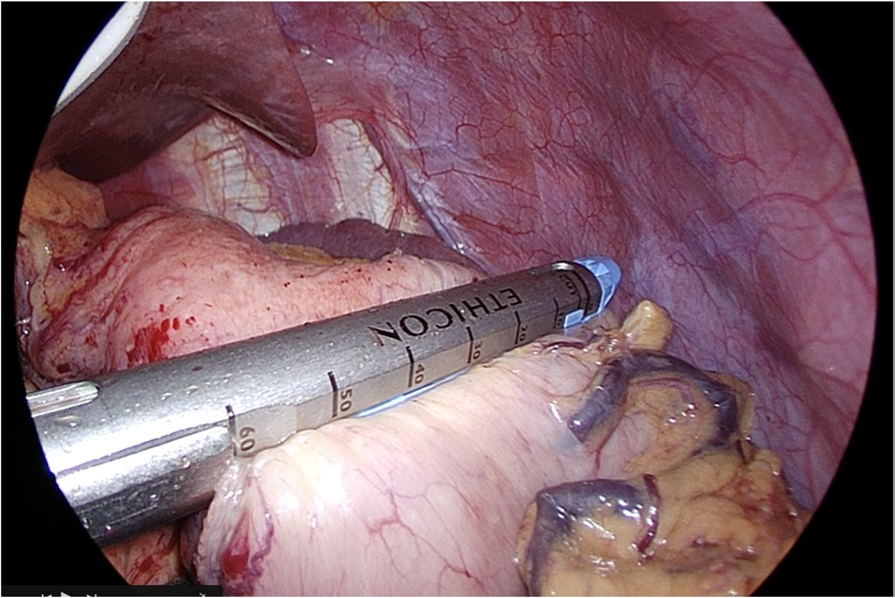
Surgical procedure: stapling the proximal side of the tumor. The second 60-mm stapler was shot to preserve the greater curvature side as much as possible

**Fig. 5:**
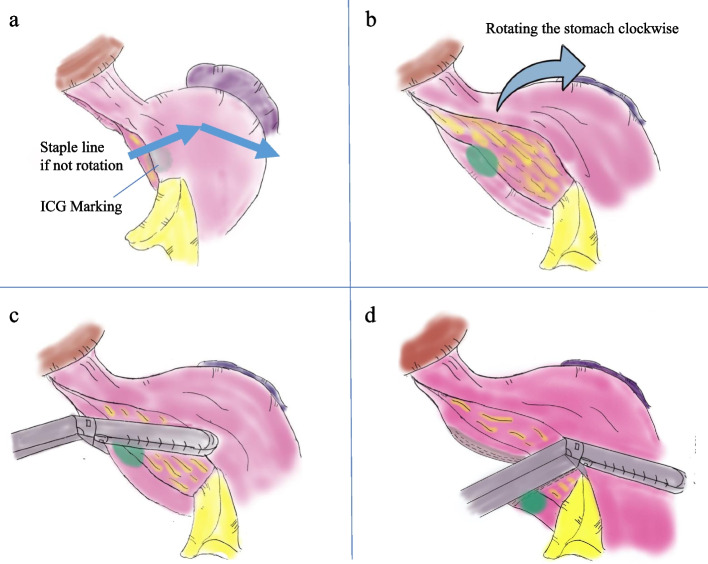
Shema of surgical procedure. **a** The tumor was located on the posterior wall of the upper body of the stomach. **b** The tumor in the posterior wall was fixed in the lesser curvature by rotating the stomach clockwise. **c** A 60-mm stapler was used from the lesser curvature side. **d** The second 60-mm stapler was stapled to preserve the greater curvature side as much as possible

An IR laparoscope camera (VISERA ELITE II, OLYMPUS, Japan) was used to confirm the tumor position. The tumor was located on the posterior wall of the upper and middle body of the stomach (Fig. 3a). The tumor in the posterior wall was fixed in the lesser curvature by rotating the stomach clockwise preserving the residual stomach as much as possible (Fig. 3b, c). It was decided to staple the right side from the lesser curvature to the greater curvature. A 60-mm stapler (Powered Echelon Flex GST system Blue 60 mm; ETHICON Endosurgery, Cincinnati, OH) was used from the lesser curvature side to ensure a 2-cm margin on the tumor’s proximal side (Fig. 3d). The same 60-mm stapler, as the second shot, was stapled to preserve the greater curvature side as much as possible (Fig. 4). The resected specimen was removed through the umbilical incision. Figure 5 shows this surgical procedure as a shema.

#### Reconstruction

**Fig. 6:**
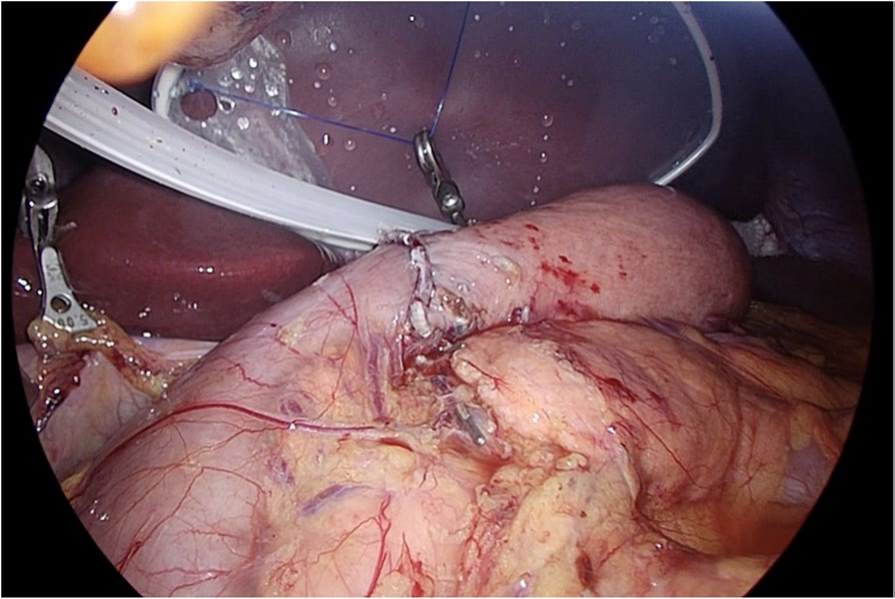
Billroth I reconstruction. Billroth I reconstruction using the delta-shaped anastomosis

A delta check was passed as the duodenum’s posterior wall, and the remnant stomach’s posterior wall was sufficiently mobilized. B-I reconstruction using the delta-shaped anastomosis was performed (Fig. 6).

### Postoperative course

**Fig. 7:**
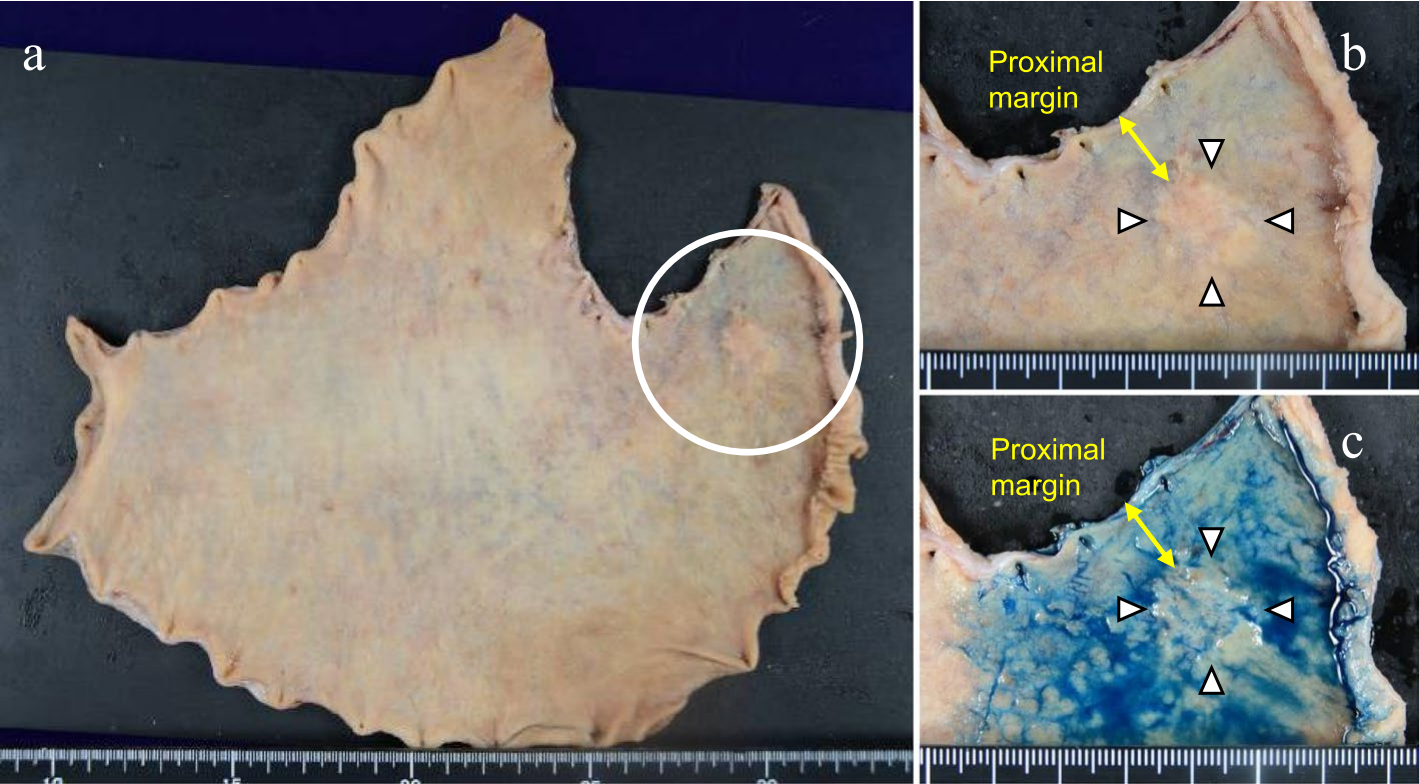
Surgical specimen. **a** White circle shows the tumor. **b** White arrow shows the tumor. Proximal margin is showed by yellow narrow. **c** Tumor dyed with indigo carmine

The operation time was 234 min, and the intraoperative blood loss was 5 ml. The patient was allowed to be discharged on postoperative day 6 without complications. Histology reported a gastric carcinoma with lymphoid stroma, T1b2, INFb, Ly0, V1c, Pn0, PM0, RM0, and N0 (0/36) (Fig. 7). Six months after surgery, there are no reflux esophagitis and weight loss due to poor appetite.

## Discussion and conclusions

Laparoscopic proximal gastrectomy (LPG) and laparoscopic total gastrectomy (LTG) have increased in recent years for upper gastric cancer [1–4]. Total gastrectomy is often selected for safer R0 resection in advanced upper gastric cancer. However, it is difficult to determine the extent of the resection margin and reconstruction method in terms of postoperative gastric function in upper and middle early gastric cancer. LTG and LPG are technically more challenging than LDG. Hiki et al. reported the feasibility of laparoscopy-assisted subtotal gastrectomy (LAsTG) for these problems [5, 6]. Previous LAsTG studies have chosen RY reconstruction in all cases, but RY reconstruction has the complexity of the complete intracorporeal operation and was reported to increase postoperative complications [7]. Then, we selected B-I reconstruction for patients with minimal remnant stomach to simplify the procedure, shorten the operation time, and reduce postoperative complications. There are some minimal gastric resection or RY reconstruction by LAsTG studies in the past. Still, no B-I reconstruction reports in the minimal gastric remnant have been accepted.

When performing anastomosis in the minimal residual stomach for upper and middle gastric cancer, care must be taken in determining tumor location to secure the oral side tumor margin. We believe ICG fluorescence marking is the most helpful method for accurately recognizing tumor location and choosing the surgical resection line [8, 9]. As in the present case, even when the tumor location is complicated, visualization of the tumor location for all surgeons greatly simplifies the stapling procedure.

It is necessary to devise a stapling technique method for the oral side after accurately determining the tumor position for gastric cancer on the upper and middle body posterior wall. In our case, the tumor portion was fixed from the posterior side to the lesser curvature side by mobilizing and rotating the stomach clockwise. Stapling was performed in two planned sessions from the lesser curvature to the greater curvature. This rotation method has some advantages. First, the tumor side must be reliably separated at the first stapling to ensure oncological safety, where the tumor margin is most critical. Second, the second stapling should be able to preserve the remaining greater curvature side as much as possible and secure a residual stomach that will allow for B-I reconstruction.

Posterior duodenal wall and posterior remnant gastric wall mobilization is safe and useful as a point that enables B-I reconstruction with delta-shaped anastomosis in the minimal residual stomach. Mobilization and less tension between the residual stomach and duodenum would reduce anastomotic leakage and other problems.

## Conclusion

The LDG and B-I reconstruction indication could be expanded to cases where LTG or LDG and RY reconstruction has been selected for early-stage gastric cancer in the upper and middle gastric body using ICG marking and gastric rotation method.

## Supplementary Information

**Additional file 1.** Surgical procedure. Laparoscopic distal gastrectomy and D1+ lymphadenectomy with indocyanine green marking and a method of rotating the stomach for stapling.

## Data Availability

Not applicable.

## Abbreviations

ICG: Indocyanine green
B-I: Billroth I
LDG: Laparoscopic distal gastrectomy
EGJ: Esophagogastric junction
LPG: Laparoscopic proximal gastrectomy
LTG: Laparoscopic total gastrectomy
LAsTG: Laparoscopy-assisted subtotal gastrectomy
